# Understanding the need of ventricular pressure for the estimation of diastolic biomarkers

**DOI:** 10.1007/s10237-013-0531-y

**Published:** 2013-10-04

**Authors:** Jiahe Xi, Wenzhe Shi, Daniel Rueckert, Reza Razavi, Nicolas P. Smith, Pablo Lamata

**Affiliations:** 1 Department of Computer Science, Oxford University, Oxford, UK; 2 Department of Computing, Imperial College London, London, UK; 3 Department of Biomedical Engineering, St Thomas Hospital, King’s College London, London, UK

**Keywords:** Cardiac computational modeling, Parameter estimation, Diastolic biomarkers, Myocardial stiffness, Residual active tension

## Abstract

The diastolic function (i.e., blood filling) of the left ventricle (LV) is determined by its capacity for relaxation, or the decay in residual active tension (AT) generated during systole, and its constitutive material properties, or myocardial stiffness. The clinical determination of these two factors (diastolic residual AT and stiffness) is thus essential for assessing LV diastolic function. To quantify these two factors, in our previous work, a novel model-based parameter estimation approach was proposed and successfully applied to multiple cases using clinically acquired motion and invasively measured ventricular pressure data. However, the need to invasively acquire LV pressure limits the wide application of this approach. In this study, we address this issue by analyzing the feasibility of using two kinds of non-invasively available pressure measurements for the purpose of inverse mechanical parameter estimation. The prescription of pressure based on a generic pressure–volume (P–V) relationship reported in literature is first evaluated in a set of 18 clinical cases (10 healthy and 8 diseased), finding reasonable results for stiffness but not for residual active tension. We then investigate the use of non-invasive pressure measures, now available through imaging techniques and limited by unknown or biased offset values. Specifically, three sets of physiologically realistic synthetic data with three levels of diastolic residual active tension (i.e., impaired relaxation capability) are designed to quantify the percentage error in the parameter estimation against the possible pressure offsets within the physiological limits. Maximum errors are quantified as 11 % for the magnitude of stiffness and 22 % for AT, with averaged 0.17 kPa error in pressure measurement offset using the state-of-the-art non-invasive pressure estimation method. The main cause for these errors is the limited temporal resolution of clinical imaging data currently available. These results demonstrate the potential feasibility of the estimation diastolic biomarkers with non-invasive assessment of pressure through medical imaging data.

## Introduction

An increasingly important research area within the field of cardiac modeling is the development and study of methods of model-based parameter estimation from clinical measurements of cardiac function (Wang et al. 2009, 2010; Moireau and Chapelle 2011; Chabiniok et al. 2011; Xi et al. 2013, 2011). This set of techniques provides an comprehensive approach to the quantification of cardiac function, with the potential for improved selection of individuals with pathological myocardial mechanics for further therapy (Nagel and Schuster 2010). In organ-level cardiac mechanical models, both passive constitutive material parameters and active contractility parameters are required for the simulation of diastolic and systolic functions, respectively (Niederer and Smith 2009; Nordsletten et al. 2011). As such, these active and passive parameters are important physiological variables related to the function of the heart.

In particular, the diastolic function (i.e., left ventricular-LV—blood filling) is affected by two main characteristics of the myocardium (Zile et al. 2004; Maeder and Kaye 2009): (1) its capacity to relax, produced by the release of the actin-myosin cross-bridges, and (2) its compliance (or its reciprocal, stiffness) often quantified within models via constitutive material parameters that dictate the capacity of the LV chamber to passively dilate. These two physiological properties, corresponding to the diastolic residual active tension (AT) and passive constitutive parameters in the mechanical model, are difficult to assess in vivo. For this reason, to a significant degree, the traditional criterion to diagnose diastolic dysfunction is subject to many limitations and controversies (Maeder and Kaye 2009).

In our previous work (Xi et al. 2013), we demonstrated the feasibility of estimating these diastolic mechanical parameters and decoupling the effects of active recoil and passive inflation. Using a model-based approach, a clear difference was shown in the diastolic mechanical parameters (i.e., the stiffness and diastolic residual active tension) between healthy and diseased subjects. However, the need for both motion and pressure measurements in a single subject to utilize this method limits its wide application. Specifically, accurate values of LV pressure are only available in the clinic via an invasive cardiac catheterization procedure, where a catheter is typically introduced through a femoral artery and advanced to the LV (Sasayama et al. 1984; Urheim et al. 2002; Zile et al. 2004). Furthermore, while the cardiac catheterization remains the clinical standard, these measurements can be affected by calibration errors (Solomon and Stevenson 2009), and the potential complications and health risks associated with this invasive technique underscore the need for reliable non-invasive methods to measure LV pressures (Chatterjee 2009; Solomon and Stevenson 2009).

As a potential alternative to invasively acquired data, there are three main non-invasive methodologies for estimating the LV diastolic pressure. The first approach uses 4D velocity fields available via specialized US, CT or MR imaging protocols and then computes the pressure gradients by solving the Navier Stokes equation, typically with a number of simplifications (Krittian et al. 2012; Song et al. 1994; Yotti et al. 2011). This method can potentially provide pressure maps with high spatio-temporal resolution (Pitcher et al. 2013), but pressure values can only be computed relative to one point in that domain. Therefore, absolute values are not available without a known reference. This technique has been successfully applied to compute LV filling pressure gradients (Ebbers et al. 2001; Yotti et al. 2011) and thus has the potential to also be used for the estimation of diastolic parameters. The second methodology uses a microbubble-based ultrasound contrast agent (UCA) and is based on the fact that the change in the acoustic properties of UCA depends on blood pressure (Forsberg et al. 2005; Dave et al. 2012). The use of UCA has been approved in the United States for clinical LV opacification studies (Dave et al. 2012) and has the potential to non-invasively monitor LV pressures in real time, with reported pressure offset errors ranging from 0.025 to 0.33 kPa (Dave et al. 2012; Geoffrey et al. 2003). With both methods, LV pressure can effectively be estimated in relative terms, with an uncertain amount of offset in its absolute value. A further approach to the central blood pressure estimation is to non-invasively measure radial artery pressures, from which a transfer function is applied, as introduced by (Karamanoglu et al. 1993). While this approach has been widely used in the last few years for central pressure estimation (Hope et al. 2008), as currently implemented, it is not valid for the assessment of diastolic LV pressure, since a closed valve isolates aortic and ventricular domains during diastolic filling.

The aforementioned developments in LV pressure measurement technology present both challenges in the relative nature of the resulting data or errors in pressure offset and opportunities for estimation of mechanical properties. In this context, this study addresses two important questions for the clinical translation of techniques that enable the estimation of passive stiffness and active tension parameters: are pressure measurements required for parameter estimation? If so, what is the impact of the presence of errors in the pressure offset value required to transform the relative pressure measures into absolute values? A parameter estimation methodology robust to offset errors will enable the use of non-invasive pressure methods, such as UCA measurements (Geoffrey et al. 2003; Forsberg et al. 2005; Dave et al. 2012) or relative pressure fields from velocity data (Krittian et al. 2012; Song et al. 1994; Yotti et al. 2011).

The investigation of the importance of the LV pressure boundary condition on the problem of estimation of diastolic proprieties is developed as follows. We analyze two methods to prescribe pressure boundary conditions, i.e., to impose the absolute value of pressure (assumed constant through the ventricle) at any time during diastolic filling, in our previously reported estimation methodology (Xi et al. 2013). Firstly, we investigate the reliability of prescribing pressure based on a LV pressure–volume relationship widely accepted in literature. Using this approach, we show that this generic relationship adds little to our ability to discriminate between healthy and diseased cases, when compared with the information extracted only from images. Secondly, we investigate the use of pressure with an unknown or biased offset by analyzing results on three sets of physiologically realistic synthetic measurements with three levels of diastolic residual active tension as benchmarks. The quantification of errors against possible pressure offsets within physiological limits will lead us to determine the feasibility of this second approach.

## Materials and methods

### Method for the estimation of diastolic biomarkers

The estimation of diastolic biomarkers is based on the solution of an inverse problem, where the stiffness and decaying active tension parameters are found to best explain the relationship between the deformation and pressure of the left ventricle during diastole. A 1D spring model with discrete measurement points is used to explain the key concepts and assumptions underlying this methodology, and the reader is referred to (Xi et al. 2013) for a detailed description.

**Fig. 1:**
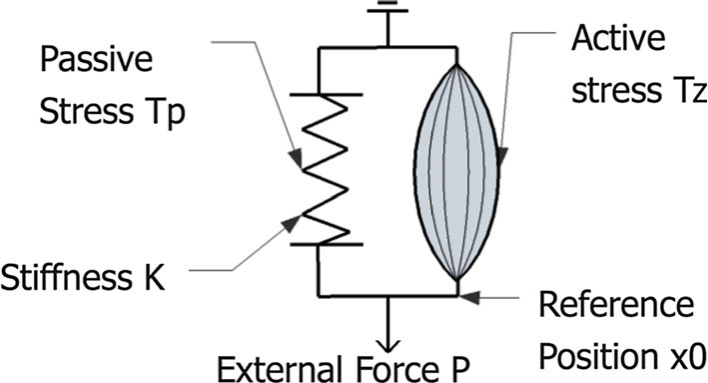
Schematic representation of the LV mechanics with a 1D spring model similar to the one used in (Remme et al. 2011): The displacement of the spring $x$, relative to the reference position $x_0$, is driven by both the external loading force $P$ and the active stress $T_z$. The passive stress $T_p$ is developed as the spring is deformed, relating to the spring stiffness constant $K$

As shown in Fig. 1, the deformation of the spring (analogous to the deformation of LV) is driven by two factors: stretching by the external force $P$ (analogous to the LV cavity pressure increasing during venous return) and contracting by the active stress $T$ (analogous to the active tension developed by the contraction of the myocardial fiber). Passive stress $Tp$ is developed when the spring is stretched or compressed from the reference position $x_0$ to another position $x_i$. Stress and deformation are assumed to be related linearly through the spring constant $K$ (stiffness) in this simplified illustrative model (note that a nonlinear relationship is actually used in the 3D model).

**Fig. 2:**
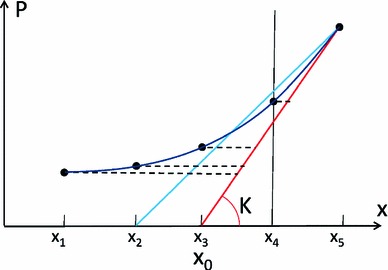
Illustration, in a pressure-displacement (P-x) curve, of the parameter estimation process. The result of a correct estimation of both stiffness $K$ and reference position $x_0$ is represented by drawing a tangent line (*red line*) for the P-x curve at the final measurement point, reaching the *horizontal line* of $P=0$ (essentially analogous to deflating from the end diastolic state to zero pressure for the LV model). The amount of active tension is proportional to the length of the 5 *horizontal dashed lines* at the 5 measurement points, because that is the amount of force needed to compress the spring from the *red line* (pure passive behavior) to the corresponding positions of the P-x curve. The *blue line* represents the scenario of an incorrect estimation of the reference position, where the AT estimated at measurement point 4 (denoted by the *dash vertical line*) will be negative (note that the *blue line* falls to the left of the P-x curve, i.e., negative AT is needed to stretch the spring to match the P-x curve)

Measurements available in the clinic during diastole are typically 5–6 image frames by dynamic magnetic resonance studies, which define the deformed position $x_i$, and a pressure recording to impose the external loading force $P_i$. The ’unknowns’ of the system are $T_z$ at each frame, $K$ and $x_0$. The solution of this problem is possible with two additional assumptions: The remaining active tension at the end of diastole is nominal, and the reference configuration $x_0$ is similar to one point during the diastolic sequence. The first assumption reduces by one the number of variables to estimate and is justified by measurements of active relaxation time (average time for full relaxation of 122.5 and 206.5 ms for controls and heart failure cases, respectively Zile et al. 2004). The second assumption was introduced to challenge the convention of taking the frame with minimum pressure as the reference frame (Wang et al. 2009) and is used to find the configuration when the active contractile force is balanced with the external inflating pressure applied to the ventricle (equivalent to the definition of the reference volume in Remme et al. 2011, when the passive forces are null). With the second assumption, $x_0$ can be estimated as the first position that leads to a continuing decaying profile of active tension, without negative values, as illustrated in Fig. 2.

### Estimation using literature P–V relationship

In order to apply our model-based parameter estimation methodology to clinical cases without invasive pressure recordings, we require a method to infer pressure from commonly available measurements (i.e., MRI). In literature, LV pressure–volume (P–V) data have been reported extensively (Kawaguchi et al. 2001; Steendijk et al. 2006; Brinke et al. 2010), and the LV P–V relationship has been well studied (Zile and Brutsaert 2002; Zile et al. 2004; Klotz et al. 2007). Thus, for the purpose of parameter estimation without pressure information, we first consider the implications of inferring the LV pressure using relationships defined from this data.

#### $P_Z$: LV pressure surrogate based on Zile et al. (2004)

The diastolic LV P–V relationship has previously been described by an exponential equation (Zile and Brutsaert 2002; Zile et al. 2004), $$P=Ae^{\beta V}$$, where $$P$$ is the left ventricular diastolic pressure, $$V$$ is the left ventricular diastolic volume, and $$A$$ and $$\beta $$ are empirically determined constants used to quantify passive stiffness. This empirical P–V relationship has been widely used, and the stiffness constant $$\beta $$ is currently the gold-standard for the characterization of the stiffness of the heart in the clinical practice (Aurigemma and Gaasch 2004; Burkhoff et al. 2005; Westermann et al. 2008). To understand the impact of pressure on the parameter estimation, we use the P–V relationship ($$P=2.3 e^{0.01V})$$ measured in the control cases (Zile et al. 2004) for the following experiments. The reason for this choice is that, using this single pressure profile prescribed in the same way (i.e., without biasing estimation results by the prescribed pressure), our goal is to analyze, as a criteria of assessing the plausibility of estimated parameters, how the estimation of AT and stiffness differs between healthy and diseased subjects.

It is important to notice that the P–V relationship chosen was defined by removing the effect of the active recoil from the pressure transient and fitted to three points during diastole: (1) minimum volume, (2) point prior to atrial kick and (3) end-diastolic volume (Zile et al. 2004).

**Fig. 3:**
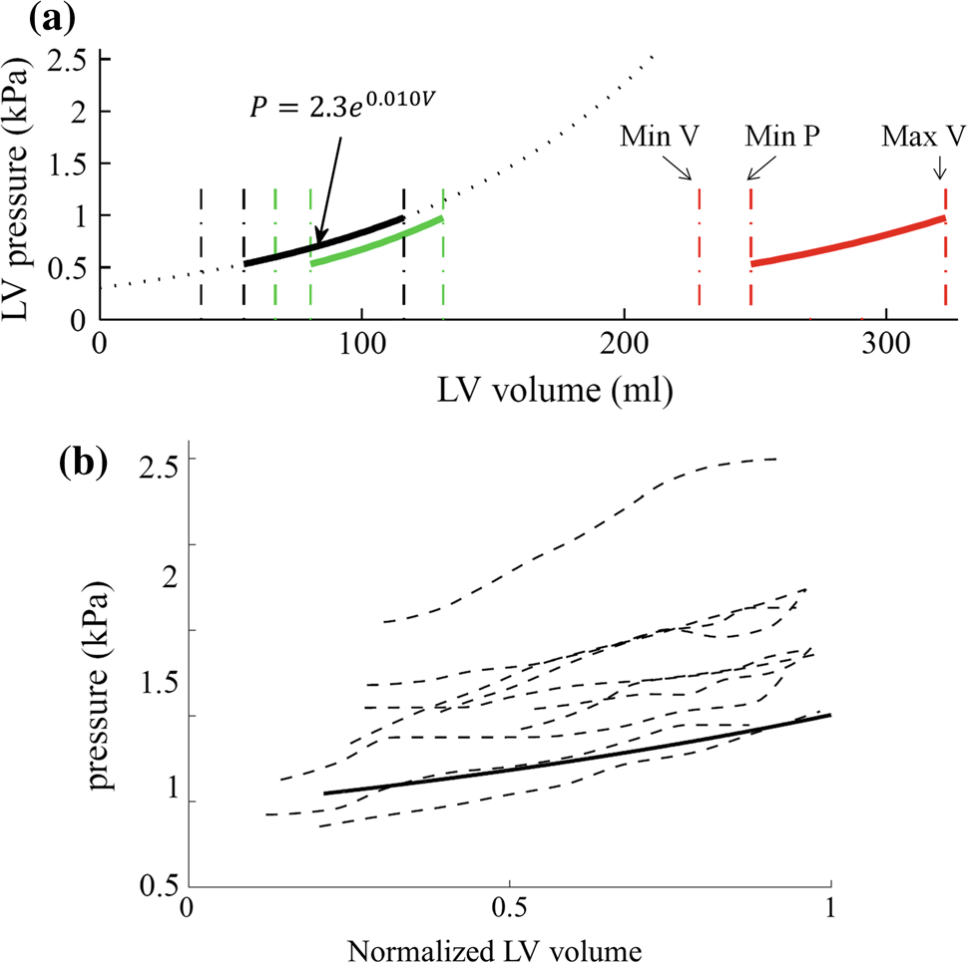
Pressure surrogate $P_Z$ using a normalized diastolic P–V relationship reported in (Zile et al. 2004). **a** Direct application of P–V relationship reported in (Zile et al. 2004) (*black sold line*, direct extrapolation to large volume ranges, represented in *dashed font*, will lead to not physiological pressure values) and $P_Z$ computed for two cases after volume normalization (*green* and *red lines*, details in Sect. 2.2). **b** Comparison of $P_Z$ (*solid line*) against diastolic P–V data (*dash lines*, the same volume normalization is performed) reported in literature for heart failure patients with large LV volumes

It is also important to note that the P–V relationship cannot be used directly, because when it is applied to diseased hearts with a large LV volume, this relationship will produce unrealistic pressure (Fig. 3a). To address this limitation, we use the normalized volume as suggested by (Klotz et al. 2007). As shown in Fig. 3a, this normalization is done using the diastolic LV minimum and maximum volume (denoted by the black leftmost and rightmost vertical dashed lines). To infer the LV pressure for new cases (the blue and red curves in Fig. 3a), the same normalization is undertaken on each of the new cases to get the normalized volume. Pressure is then determined by indexing the normalized P–V relationship. In Fig. 3b, we verify this normalization-based P–V relationship by comparing it to the diastolic P–V data reported in literature for AHA class II and III patients (Lorusso et al. 1997; Kawaguchi et al. 2001; Steendijk et al. 2006; Brinke et al. 2010).

#### Application of LV pressure surrogate to 18 clinical cases

Using the method of a literature-based pressure value $P_Z$, we apply the parameter estimation methodology proposed in our previous work (Xi et al. 2013) to a total of 18 clinical cases with only imaging data. As introduced earlier, we analyzed the parameter difference between healthy and diseased cases and compared these results to the information extracted only from the imaging measurements.

**Fig. 4:**
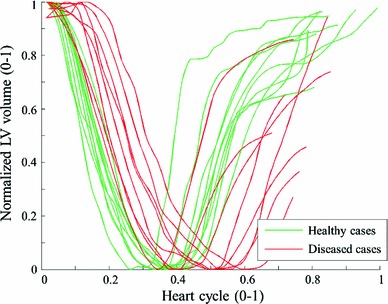
Normalized volume transients over a heart cycle for the 18 clinical cases

The imaging data are short axis cine MRI acquired in St Thomas’ Hospital London. The data sets used in the study conform to the principles outlined in the Declaration of Helsinki, and the study was carried out as part of a local ethics committee approved protocol with informed consent obtained from the subjects. In these 18 cases, 10 cases are healthy volunteers and 8 are heart failure patients. A summary of each case is provided in Table 1. The LV volume transients (normalized) are plotted in Fig. 4 which, interestingly, shows a clear difference in the timing of the minimum volume points, suggesting a delayed diastolic relaxation for the diseased cases.

**Table 1:** Patient information of the 18 clinical cases

	Sex	Age	HR	Wt	EF	$V_{max}$	AHA
H1	M	34	74	72	49	131	0
H2	M	32	77	82	44	110	0
H3	M	27	50	89	49	193	0
H4	M	29	61	65	41	165	0
H5	M	22	67	65	45	118	0
H6	M	22	69	73	40	143	0
H7	M	30	54	74	36	160	0
H8	M	31	71	65	38	153	0
H9	F	24	50	54	54	156	0
H10	M	20	74	85	43	204	0
D1	M	79	43	70	26	365	3
D2	M	66	83	85	18	318	3
D3	F	65	57	65	19	328	2
D4	M	62	57	110	23	282	3
D5	M	80	62	90	30	243	3
D6	M	58	54	90	33	353	2
D7	M	58	77	104	26	225	2
D8	F	76	59	54	30	204	3

H stands for healthy, and D stands for diseased. *HR* heart rate (beats per min), *Wt* weight (kg), *EF* ejection Fraction, $V_{max}$ maximum LV volume (ml), *AHA* American heart association classification of heart failure (from 0 to 4)

The parameter estimation methodologies, including the methods for processing the MR measurements, mechanical model simulation and algorithm for estimating the AT and material properties, are described in detail in (Xi et al. 2013). In brief, the cine MRI sequence is first processed using an motion tracking algorithm (Shi et al. 2012) to extract the myocardial displacements, based on which a sequence of cubic-Hermite meshes are then constructed and aligned to the motion observed in each frame of MRI sequence (Lamata et al. 2011). These meshes are compared to the simulation results generated using our finite deformation-based mechanical model, with the previously defined pressure surrogate (illustrated in Fig. 3d) as its boundary condition. The mechanical parameters are then estimated using our novel parameter algorithm (outlined in 2.1). It is important to note that, because the cine MRI data do not provide accurate 3D displacements within the myocardium, the parameter optimization criterion is based in this study on LV volume (an integral metric) instead of 3D displacements (a detailed description of strain).

### Parameter estimation with unknown or biased LV pressure offset

Our second goal in this study is to assess the accuracy of parameter estimation using pressure measurements with unknown or biased offset values. In order to achieve this goal, synthetic cases with ground-truth values provide a clean set of benchmarks to quantitatively analyze the error introduced with the presence of pressure offsets. These synthetic recordings represent idealized non-invasive pressure data from imaging methods (such as UCA Geoffrey et al. 2003; Forsberg et al. 2005; Dave et al. 2012 or relative pressure fields from velocity data Krittian et al. 2012; Song et al. 1994; Yotti et al. 2011). Note that the scope of this work is not to provide a complete methodological pipeline from image measurements to diastolic biomarkers, but to assess the feasibility and potential of using non-invasive pressure estimation methods.

**Fig. 5:**
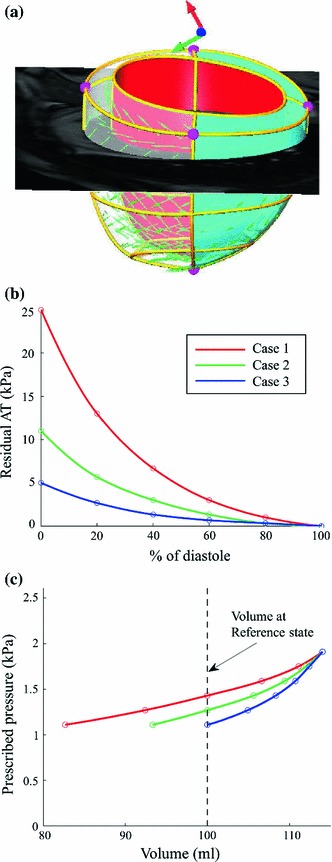
Illustration of the generation of three synthetic measurements used for parameter estimation experiments. **a** Stress-free reference geometry (visualized with one slice of short axis cine MRI), a patient-specific geometrical model constructed from MR images (Xi et al. 2013). **b** Three types of decaying residual active tension profiles, which are prescribed in the synthetic simulations to present three levels of disease conditions (i.e., impaired relaxation during early diastole). **c** Volume of the six simulated synthetic measurements (six states during diastole) for each case, together with the corresponding prescribed LV pressure. Note that since the reference geometry and constitutive parameters are assumed to be the same for the three synthetic cases and the residual tension is zero at end-diastole, the simulated end-diastolic volume is thus the same

Synthetic measurements are simulated using our LV mechanical model. The reference geometrical model, with patient-specific geometry published in (Xi et al. 2013), is shown in Fig. 5a. A set of typical Guccione constitutive parameters ($C_1=1, C_2=30, C_3=20, C_4=20$) is prescribed, and three types of decaying residual active tension were simulated, respectively, representing different levels of diseased conditions with impaired LV relaxation capabilities during diastole (Fig. 5b). The pressure range (Fig. 5c), from the beginning of diastole pressure (BDP) to the end of diastole pressure (EDP), is prescribed as 1.1–1.9 kPa, based on the average values of AHA class II and III patients reported in literature (Lorusso et al. 1997; Kawaguchi et al. 2001; Steendijk et al. 2006; Brinke et al. 2010). Six measurements were simulated evenly distributed in time, which is the typical number of MRI frames covering the diastole phase. Note that our method relies on the assumption that at one measurement point, the active contractile stress and passive inflating pressure are roughly balanced: Under the prescribed levels of AT in our simulation, the volume at 3rd, 2nd and 1st measurements points for cases 1, 2 and 3, respectively, is close to the volume of the stress-free reference geometry (i.e., the reference volume denoted by the dash line in Fig. 5c).

Parameter estimation, using the same method as applied to the cases in Sect. 2.2, is performed using each of the three sets of synthetic measurements.

We performed the parameter estimation with eight evenly distributed values of pressure offset ($-$1.10, $-$0.73, $-$0.36, 0, 0.39, 0.76, 1.13, 1.50 kPa), which shift the BDP from a minimum of 0 kPa to a maximum of 2.6 kPa, corresponding to the physiological range of BDP reported in literature (Lorusso et al. 1997; Kawaguchi et al. 2001; Steendijk et al. 2006; Brinke et al. 2010).

## Results

We present the results of parameter estimation either using the literature-based pressure surrogate ($P_Z$) or introducing pressure offset errors in the following two subsections.

### Feasibility of parameter estimation without any pressure data

**Fig. 6:**
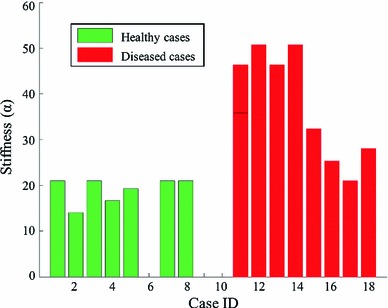
Estimated $\alpha$ (stiffness) of the Guccione material parameters for the 18 clinical cases with $P_Z$: Cases 1–10 are healthy and cases 11–18 are diseased. Due to the convergence issue, 3 of the 10 healthy cases could not be simulated

Figure 6 plots the $\alpha$ values (stiffness, defined in Xi et al. 2013) of the estimated Guccione parameters for the 18 clinical cases using $P_Z$. Overall, there is a significant difference between healthy and diseased cases in terms of the stiffness implied by $\alpha$, which agrees with the prior knowledge of disease classification. However, it is likely that the difference in the $\alpha$ values between the healthy and diseased cases could be already implied by the difference in the ejection fraction (EF) calculated from the volume (further details in discussion section).

**Fig. 7:**
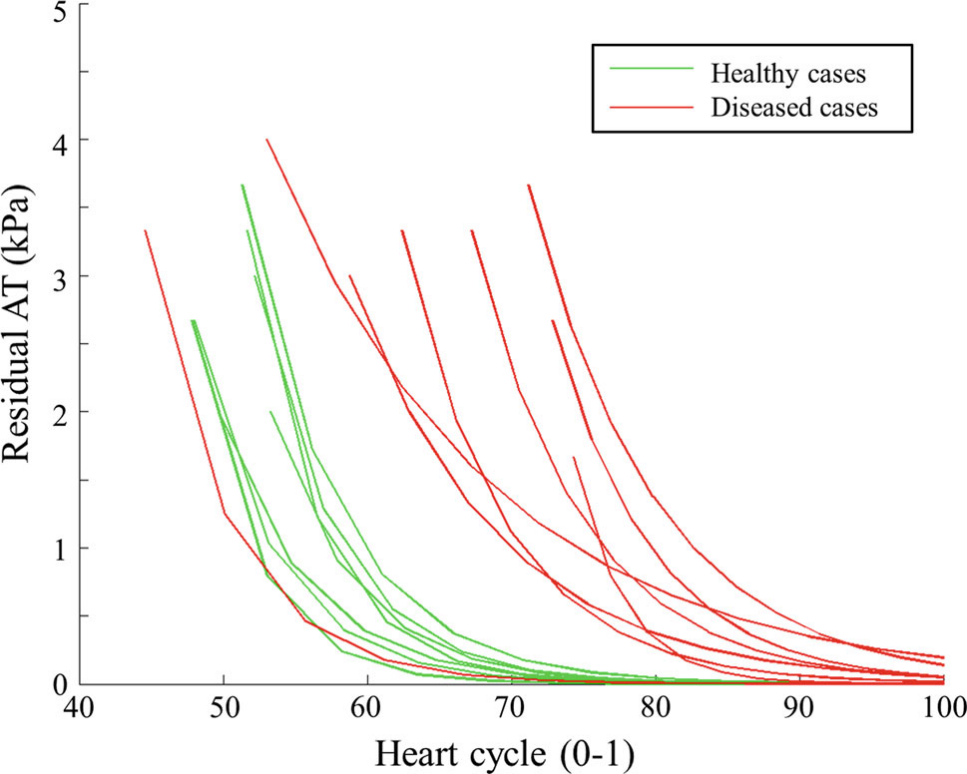
Estimated diastolic AT transient for the 18 clinical cases with $P_Z$. Transients are grouped by disease class: healthy (*green*) and diseased (*red*). As described in (Xi et al. 2013), AT transients shown here are fitted exponential curves

Figure 7 plots the estimated diastolic AT transients for the 18 clinical cases using $P_Z$. The AT transients are quite similar across the healthy and diseased cases, which is inconsistent with disease classification. The difference in the timing of AT can be explained by the volume transient in Fig. 4, which shows the different timing at the beginning of diastole. Notably, although the deformation of the 18 clinical cases are significantly different (in terms of the LV volume and ejection fraction), surprisingly the estimated AT transients (especially in terms of the maximal AT) are similar, indicating that AT is likely to be highly coupled with the prescribed pressure. This difference in the estimated AT transients between healthy and diseased cases is significantly smaller then that reported in (Xi et al. 2013) using the measured LV pressure.

### The impact of pressure offset errors in parameter estimation

**Fig. 8:**
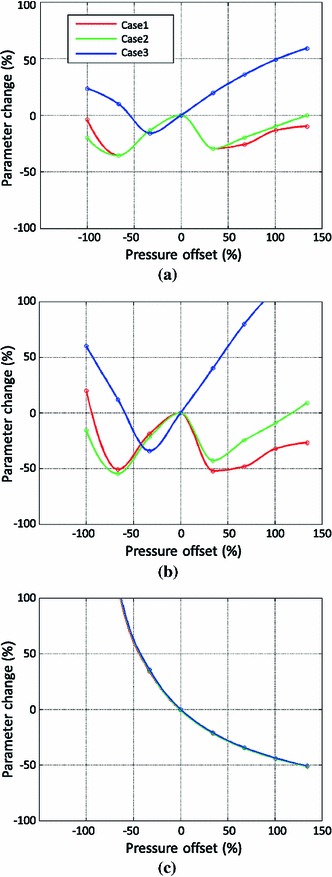
The percentage change of estimated parameters [$\alpha$ (**a**) and AT (**b**)] with respect to the percentage offset of the LV pressure, comparing to the percentage change of clinical stiffness index $\beta$ (Zile et al. 2004) in (**c**). The percentages are calculated using the ground-truth parameters prescribed in the three synthetic cases. These results are explained in details in Sect. 3.2

Figure 8 shows, using the three sets of synthetic benchmark cases, the percentage change of estimated parameters–$\alpha$ of Guccione parameters (Fig. 8a), maximal diastolic AT (Fig. 8b)—with respect to the percentage offset of relative pressure with respect to the beginning of diastole pressure (1.10 kPa) from $-$100 to $+$136 % (or $-$1.10, $-$0.73, $-$0.36, 0, 0.39, 0.76, 1.13 and 1.50 kPa in their absolute values, $-$100 % corresponding to an offset of $-$1.10 kPa and $+$136 % to an offset of $+$1.50 kPa). For further reference, the traditional empirically derived clinical stiffness index $\beta$ is calculated for all these experiments following the guidelines (Zile et al. 2004) and compared in Fig. 8c to the stiffness parameter $\alpha$ calculated using our model-based methods.

Overall, the changes in $\alpha$ and AT are not monotonically increasing/decreasing until the pressure offset reaches a positive limit (i.e., shifted up to a limit). This limit is first reached in case 3 (from 0 %), where the AT is the smallest among the three cases. This is because the 1st measurement is already close to the reference state, and a positive pressure offset would cause the estimated reference state to go beyond the 1st measurement (as explained in detail in the discussion section).

When the pressure offset is negative (i.e., pressure shifted downwards), $\alpha$ changes by a maximum amount of 35 % (in contrast, $\beta$ changes by $-$51 to $+$1120 %), and AT by a maximum of 60 %. The maximal error ranges for $\alpha$ and AT also hold for the positive pressure offset, but not after the monotonically increasing limit, after which the estimated parameter will increase without an upper bound.

In the $\pm$30 % window of pressure offset, which corresponds to the reported maximal measurement error 0.33 kPa using the current state-of-art non-invasive pressure estimation method (Dave et al. 2012), the maximal changes are 27 % for $\alpha$ and 45 % for AT, respectively. In the $\pm$16 % window of pressure offset, which corresponds to the reported mean error (0.17 kPa) in the pressure estimation, the maximal changes become 11 % for $\alpha$ and 22 % for AT, respectively. It is important to note that based on the published results in (Xi et al. 2013), the healthy and diseased cases have differences of approximately 42 % in $\alpha$, and 69 % in AT. As such, under the mean error assumption, these results show promise for delineating the healthy and diseased cases using pressure obtained from current imaging-based non-invasive estimation methods.

## Discussion

We have performed the first study, to the best of our knowledge, to analyze the feasibility of using literature-based pressure surrogates, and the impact of pressure offset errors, in the problem of inverse parameter estimation in ventricular diastole. In the following, we discuss the reliability and implications of the parameter estimation results using both sources of LV pressure information.

**Fig. 9:**
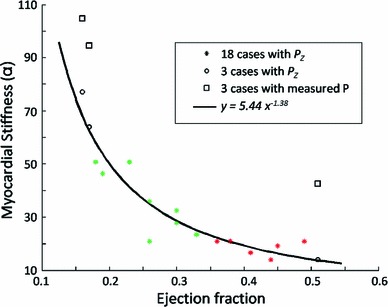
Regression line between stiffness and EF in the clinical cases (18 cine-only cases and 3 cases previously reported in Xi et al. 2013), when estimated using the literature surrogate $P_Z$. The ground-truth $\alpha$ (estimated using the measured pressure) of the three cases are plotted in *box marks*. The *green star marks* correspond to the healthy cases among the 18 cine-only cases, while the *red stars marks* correspond to the diseased ones

The stiffness estimated using a literature P–V relationship differentiated healthy and diseased cases, see Fig. 6. The relationship between ejection fraction (EF) and the $\alpha$-stiffness estimated in the previous experiments is shown in Fig. 9. When the $\alpha$-stiffness or the material properties are estimated using the same pressure surrogate $P_Z$ according to (Zile et al. 2004), they are essentially negatively correlated with the EF. Therefore, the information implied by the stiffness estimated using the prescribed pressure seems no more than a surrogate for what can be directly measured, in terms of ejection fraction, using only the data observable from the image.

Despite the significant advantage such a technique would offer in the clinic, our results show that estimation of diastolic AT and constitutive parameters without LV pressure is an ill-posed problem. Specifically, we have shown there is a clear need for both stimuli (pressure) and response (deformation) to estimate parameters of the LV mechanical system. The prescription of pressure based on a P–V relationship significantly affects the computation of AT—high similarity among the 18 clinical cases despite of clear differences in the cardiac deformation and diseased conditions, see Fig. 7. In addition, the estimated stiffness seems to be highly correlated with the ejection fraction (as discussed in previous paragraph).

**Fig. 10:**
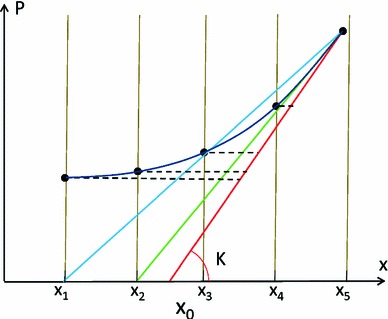
Illustration, in a pressure-displacement (P-x) curve, of the errors introduced in the parameter estimation process by an offset error in pressure data. The correct estimation of stiffness $K$ should be the *red line*, the same as the *red line* in Fig. 2. Due to the limited temporal resolution, the reference state indicated by the *red line* (between the 2nd and 3rd measurements, $x_2$ and $x_3$) is not measured, and the 2nd measurement is selected as the reference state leading to an underestimation of $K$ and AT (*green line*). Note that if the measurement point $x_4$ would not be available, the *blue line* would also fulfill the criteria of decaying not negative AT

Reported inverse modeling methodology is robust to the presence of errors in the offset of the pressure temporal transients, specifically when compared to current clinical guidelines, as shown in Fig. 8. Non-invasive pressure estimation methods may be valid for material property estimation, provided a correct handling of the limited temporal resolution in order to find a reasonable reference configuration (see Fig. 10 and further details in following paragraphs). The fundamental reason for this robustness is that our methodology will assimilate a pressure offset as a change in the estimated reference configuration (see Fig. 10 for further explanation), and this severely attenuates errors in the assessment of material properties. This result can be intuitively explained by the fact that, once the correct methodology is applied, it is an increment of pressure, not its absolute value, that causes an additional passive inflation of the tissue, and it is the ratio between these two increments that determines the stiffness of the material and the decay of active tension.

The three sets of synthetic measurements provide benchmarks to quantify the error that could be possibly introduced by the uncertainty in transforming relative pressure into the absolute values. These three synthetic cases have different levels of diastolic residual active tension covering a wide range of diseased conditions (i.e., impaired ventricular relaxation capability) in terms of the magnitude of possible residual tension (Xi et al. 2013). We adopted our assumptions for generating those synthetic benchmarks based on the averaged P–V data reported in literature (Lorusso et al. 1997; Kawaguchi et al. 2001; Steendijk et al. 2006; Brinke et al. 2010) and tested a large range of possible pressure offsets according to physiologically realistic variability of diastolic P–V data. However, we acknowledge that the experiments conducted on the in-silico cases have an inherent limitation: The measurements (both the pressure and motion measurement) are free of noise compared to the real measurements obtained in the clinic. Most often these errors are not white noise and knowing the probability distribution is challenging. Nevertheless, we believe these in-silico experiments do provide a clean and reliable estimate of the possible errors in estimated parameters using relative pressure measurements.

The main source of the errors reported in Fig. 8 is the limited temporal resolution of deformation data, which introduces a biased estimation of the reference configuration $x_0$. There are two mechanisms for it, as illustrated in Fig. 10: a *rounding error* incurred by a choice for $x_0$ among a discrete set of measurements (represented by the green line in Fig. 10), and a *differentiation error* incurred by taking the secant (defined by the criteria of decaying not negative AT) and not the tangent to the P-x curve (see blue line in Fig. 10). Note that when these two error mechanisms are correctly accounted for, $K$ and $AT$ will be correctly computed due to the fact that $x_0$ will be compensating the presence of an error in pressure offset, as explained previously.

These considerations have important implications for the design of a clinical protocol for the characterization of diastolic biomarkers. An increment in the temporal resolution during acquisition of imaging data or techniques for an adequate temporal interpolation between measurement points, become critical in order to minimize error against the presence of an unknown pressure offset. It is also important to note that the presence of remaining decaying active tension in the late phase of diastole should only be expected in cases of extreme diseased conditions.

Our results suggest that diastolic parameter estimation is feasible with non-invasive pressure data subject to pressure offset errors, such as UCA measurements (Geoffrey et al. 2003; Forsberg et al. 2005; Dave et al. 2012) or relative pressure fields from velocity data (Krittian et al. 2012; Song et al. 1994; Yotti et al. 2011). Nevertheless, further work is needed to validate the use of velocity-derived relative pressure estimates, which requires the existence of a reference point with known or constant pressure in the pulmonary venous system in order to assess LV filling pressure.

## Conclusion

This paper presents the first study, to the best of our knowledge, that analyzes the feasibility of using literature-based and relative pressure data for the purpose of inverse diastolic cardiac parameter estimation. Patient-specific LV pressure data are required for the estimation of cardiac diastolic properties. Without it, parameters may have no added diagnostic value than that extracted from images (i.e., the stiffness is correlated with the ejection fraction). Non-invasive measurements of relative LV pressure can be used for estimating parameters, and increased temporal resolution of diastolic measurements will improve the accuracy of estimated parameters.
